# Using Zebrafish to Dissect the Interaction of Mycobacteria with the Autophagic Machinery in Macrophages

**DOI:** 10.3390/biology12060817

**Published:** 2023-06-04

**Authors:** Salomé Muñoz-Sánchez, Mónica Varela, Michiel van der Vaart, Annemarie H. Meijer

**Affiliations:** Institute of Biology Leiden, Leiden University, Einsteinweg 55, 2333 CC Leiden, The Netherlands

**Keywords:** zebrafish, macrophages, live imaging, infection, innate immunity, autophagy

## Abstract

**Simple Summary:**

Tuberculosis is a life-threatening disease caused by infection with mycobacteria. These bacteria can grow inside the cells of our immune system, particularly in macrophages, one of the first cells responding to an infection. Macrophages activate several defense mechanisms to combat the infection, but mycobacteria are notorious for escaping these mechanisms. One of the macrophage defense mechanisms is autophagy, a vital process that keeps cells in a healthy condition by clearing worn-out cell parts or microbial invaders. Stimulating autophagy is explored as a potential strategy to improve the treatment of tuberculosis, but this will require a better understanding of how mycobacteria interact with the autophagy machinery. In this study, we infected zebrafish larvae with a mycobacterial species closely related to the human tuberculosis pathogen. Zebrafish larvae are transparent and ideally suited for microscopic imaging of the early stages of the infection process. Therefore, we could observe that mycobacteria, when taken up by macrophages, reside in vesicles formed by the autophagy machinery. We found that these vesicles dynamically change shapes and that they could serve as a temporary hiding place for mycobacteria, facilitating the spreading of the infection to other tissues.

**Abstract:**

Existing drug treatment against tuberculosis is no match against the increasing number of multi-drug resistant strains of its causative agent, *Mycobacterium tuberculosis* (*Mtb*). A better understanding of how mycobacteria subvert the host immune defenses is crucial for developing novel therapeutic strategies. A potential approach is enhancing the activity of the autophagy machinery, which can direct bacteria to autophagolysosomal degradation. However, the interplay specifics between mycobacteria and the autophagy machinery must be better understood. Here, we analyzed live imaging data from the zebrafish model of tuberculosis to characterize mycobacteria-autophagy interactions during the early stages of infection in vivo. For high-resolution imaging, we microinjected fluorescent *Mycobacterium marinum* (*Mm*) into the tail fin tissue of zebrafish larvae carrying the GFP-LC3 autophagy reporter. We detected phagocytosed *Mm* clusters and LC3-positive *Mm*-containing vesicles within the first hour of infection. LC3 associations with these vesicles were transient and heterogeneous, ranging from simple vesicles to complex compound structures, dynamically changing shape by fusions between *Mm*-containing and empty vesicles. LC3-*Mm*-vesicles could adopt elongated shapes during cell migration or alternate between spacious and compact morphologies. LC3-*Mm*-vesicles were also observed in cells reverse migrating from the infection site, indicating that the autophagy machinery fails to control infection before tissue dissemination.

## 1. Introduction

*Mycobacterium tuberculosis (Mtb)*, the causative agent of tuberculosis (TB), is undeniably one of the world’s most successful pathogens. TB causes over a million deaths yearly, with 1.6 million in 2021 [1]. It remains challenging to control for many reasons, including its association with poverty, antibiotic resistance problems, and the lack of an effective vaccine. Although extensive research has been conducted, we are still far from fully comprehending the details of the host–pathogen interactions that underlie the success of infections with *Mtb* and related pathogenic mycobacterial species [2]. Understanding how mycobacteria subvert the host immune defenses is crucial for developing novel therapeutic strategies [3].

Upon infection, mycobacteria encounter macrophages, the first responders from the host immune system [2]. Macrophages phagocytose the mycobacteria bacilli to eliminate them by fusing bacteria-containing phagosomes with degradative lysosomes. The survival of mycobacteria in macrophages depends on their ability to interfere with intracellular membrane trafficking required for lysosomal delivery [4,5]. In this way, macrophages provide a niche for bacterial replication. Furthermore, blocking the phagolysosomal pathway allows the bacteria to damage the phagosomal membrane and invade the cytosol [6]. This last event causes the autophagy pathway activation, which can capture cytosolic bacteria in double-membrane vesicles (autophagosomes) that are subsequently delivered to lysosomes [7,8]. The molecular hallmark of autophagosome formation is the membrane insertion of microtubule associated protein light chain 3 (LC3), requiring the conversion of LC3-I into its lipidated form LC3-II [8]. The triggering of autophagy is crucial to keep the mycobacterial infection under control [7]. However, autophagy inhibition is also a pathogenic strategy of mycobacteria [8]. The details of the interaction between mycobacteria and the autophagy machinery remain poorly understood.

Xenophagy is a selective form of autophagy whereby autophagosomes capture bacteria via ubiquitin-mediated targeting. Xenophagy activation by *Mtb* requires the functional ESX-1 type VII secretion system, which is needed for phagosomal membrane rupture, conferring the bacteria access to the cytosol [9,10]. Extracellular bacterial DNA present in the cytosol is then detected by the host cGAS-STING pathway (cyclic GMP–AMP synthase–stimulator of interferon genes), leading to the ubiquitination of bacteria and the activation of ubiquitin receptor proteins, such as p62, also known as sequestosome-1, and NDP52 (Nuclear Domain 10 Protein 52) [11]. The interaction of ubiquitin receptors with LC3 finally delivers *Mtb* into an autophagosome [12]. Alternatively, LC3 conjugation can occur directly onto the phagosome membrane in a process named LAP (LC3-associated phagocytosis), which is thought to promote phagosome maturation [13,14,15,16]. This recruitment of LC3 to single membrane vesicles (LAPosomes) requires most but not all components of the autophagy machinery and is triggered by phagosomal ROS production and activation of V-ATPase [13,14,15,16]. LC3 can be detected in LAPosomes as early as 10 min after phagocytosis, shortly after cargo internalization, whereas LC3-decorated bacteria-containing autophagosomes can take hours to form [17,18]. *Mtb* has evolved virulence mechanisms to evade xenophagy as well as LAP, illustrating the importance of both pathways as host defense mechanisms [8,19].

*Mycobacterium marinum* (*Mm*) is closely related to *Mtb* and is a widely used model pathogen to study mycobacterial infections [20]. *Mm* is a natural pathogen of zebrafish and causes systemic disease with a mechanistic progression highly similar to TB [20]. Like *Mtb*, *Mm* relies on ESX-1-mediated virulence to permeabilize phagosomes and invade the cytosol, where it can be the subject of autophagy-mediated degradation [21,22,23]. Whether *Mm* also triggers LAP is currently unknown. In this study, we build on the well-established zebrafish model for *Mm* infection in which we previously demonstrated that the autophagy machinery is significant for host defense [21,24,25]. Zebrafish embryos and early larvae have functional macrophages and neutrophils, developing a thorough innate immune response against invading pathogens [26]. For live imaging of the autophagy response in this whole organism model, we used a transgenic line ubiquitously expressing a GFP-tagged version of LC3 [27]. A highly localized tail fin infection (TFI) method makes it possible to examine *Mm*-macrophage interactions at the early stages of infection [28]. Using the TFI method, we observed macrophage recruitment and detected LC3-positive *Mm*-containing vesicles promptly after phagocytosis, suggesting that LAP pathway activation might occur during *Mm* infection. We characterized and classified the different LC3-associations over the time course of infection. This live imaging study revealed that LC3 associations with *Mm*-containing vesicles are transient and heterogeneously present inside infected phagocytes, which can provide a niche for *Mm* during infection dissemination.

## 2. Materials and Methods

### 2.1. Zebrafish Husbandry

Zebrafish (Danio rerio, strain AB/TL) were maintained and handled adhering to the directives of the local animal welfare committee of Leiden University (License number 10612) and the standard guidelines from the Zebrafish Model Organism Database (https://zfin.org, accessed on 30 January 2023). Adult fish were crossed in a single couple allowing natural fertilization at the start of the light/dark cycle (14 h light/10 h dark). Eggs were kept in egg water (60 μg/mL Instant Ocean Sea salt in Milli-Q water) at 28 °C until reaching the desired developmental stage. Previously established transgenic lines were used *Tg(CMV:GFP-LC3)* [28], *Tg(mpeg1.1:mCherry-F)* [29].

### 2.2. Tail Fin Infection

*Mm* fluorescently labeled strains were used: Mma20 strain expressing mCherry in a pSMT3 vector [30], and *Mm* M strain expressing E2-Crimson in a pSMT3 vector [31]). Culture preparation for infection experiments was essentially as previously described [32]. Specifically, *Mm* strains were grown in Difco Middlebrook 7H10 agar (Becton Dickinson and company, Temse, Belgium) supplemented with 10% oleic acid-albumin-dextrose-catalase, OADC (Becton Dickinson and company, Temse, Belgium) 0.5% glycerol, and hygromycin. A single colony was picked and resuspended in Difco Middlebrook 7H9 medium (Becton Dickinson and company, Temse, Belgium) supplemented with 10% albumin-dextrose-catalase, ADC (Becton Dickinson and company, Temse, Belgium) and 0.05% Tween 80 (Sigma-Aldrich, Darmstadt, Germany) and hygromycin 50 μg/mL. The culture was kept statically overnight at 28.5 °C. The OD was measured at 600 nm; an OD600 of 1 corresponds to approximately 10^8^ *Mm* bacteria/mL. The bacteria were harvested at logarithmic phase by centrifuging and washing them two times in sterile phosphate-buffered saline solution (PBS). The bacteria were resuspended to the desired concentration in 2% Polyvinylpyrrolidone (PVP40) in PBS (*w*/*v*).

Microinjection needles of borosilicate microcapillary glass (Harvard Apparatus, 30003OD mm O.D. × ID mm I.D.) were made with a micropipette puller device (Flaming/Brown p97, Sutter Instruments Inc., Hofheim, The Netherlands) with the following settings: air pressure 500, heat 500, pull 110, velocity 100 and time 100. The needle was loaded with the *Mm* sample and mounted onto a micromanipulator at a 30° angle (MM-33, Sutter Instrument, Hofheim, Netherlands). The micromanipulator was positioned on a stand (, M10L magnetic stand, World Precision Instruments, Friedberg, Germany) with the needle projecting under a stereomicroscope (M50, achromat 1× objective 0.15 NA, transmitted light, Leica, Wetzlar, Germany). The needle tip was broken with fine tweezers to obtain a tip opening diameter of 2.5–5 μm. The injection settings were: injection time of 0.2 s, compensation pressure of 15 hPa, and injection pressure between 700 and 900 hPa. We obtained a drop size of 0.5 nL volume containing ~100 CFU. Zebrafish larvae were staged at 3 days post fertilization (dpf) by checking for a head trunk angle of 25° [33] and anesthetized with 200 μg/mL buffered 3-aminobenzoic acid (Tricaine, Sigma-Aldrich, Darmstadt, Germany)). Next, the larvae were pipetted onto a flat 1% agarose plate and injected between the two epidermal layers at the ventral part of the tail fin. To check for correct injection, we observed that the bacterial suspension temporarily forms a blister-like thickening before dispersing throughout the tissue.

### 2.3. Confocal Laser Scanning Microscopy (CLSM)

Immediately after microinjection, anesthetized larvae were mounted in custom-made 2% agarose mold and covered with 1% low melting point agarose. After solidifying, the mold was covered with egg water plus tricaine. The sample was then taken to a Leica TCS SP8 HyD inverted confocal microscope for live imaging. We used a 40× oil immersion objective (NA 1.3). Time-lapse videos, from a total of six zebrafish larvae, were processed and analyzed using Fiji open-source software, version 2.9.0/1.53t [34].

## 3. Results

### 3.1. Tail Fin Infection High-Resolution Live Imaging

**Figure 1:**
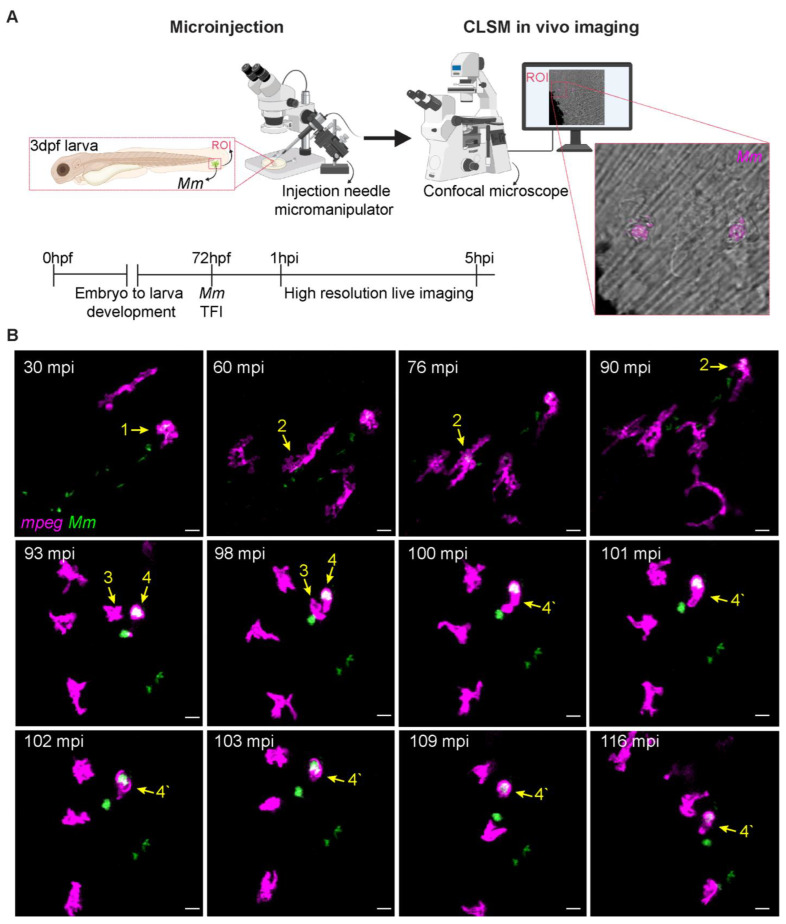
TFI high-resolution live imaging. (**A**) Schematic of *Mm* tail fin injection and imaging in zebrafish larvae (created with BioRender online website, https://www.biorender.com, accessed on 30 January 2023). Fluorescently labeled *Mm* was microinjected into the tail fin of 3 dpf larvae. Live samples were mounted in low melting point agarose and imaged. Confocal laser scanning microscopy (CLSM) live imaging was performed on the region of interest (ROI) starting from 30 mpi. (**B**) Early events of *Mm* infection in the zebrafish tail fin. Transgenic (*mpeg1.1:mCherry-F*) zebrafish larvae, where macrophages are fluorescently labeled (pseudo color magenta), were infected with 100 CFU of E2-Crimson-labelled *Mm* (pseudocolor green). Images are maximum projection stills of the first 2 h of time-lapse (Supplementary Videos S1 and S2). At 30 mpi, the first macrophage containing a bacteria cluster is observed, arrow 1. In the next 30 min, the number of recruited macrophages increases, and phagocytic events are observed, arrow 2. At 93 mpi, a macrophage (arrow 3) approaches a second macrophage containing bacteria (arrow 4), and both have formed a single cell at 100 mpi (arrow 4′). Scale bar: 10 μm.

To study the details of early events leading to a successful mycobacterial infection, we modeled this process by microinjecting *Mm* into the tail fin of zebrafish larvae at 3 days post fertilization (dpf) [28]. The experimental setup of this TFI technique (Figure 1A) makes it possible to image the interaction between bacteria and innate immune cells in real time and at high resolution. Due to the short working distance of high numerical aperture objectives used in the inverted confocal microscope, a specific mounting setup is required, where larvae are covered with low-melting agarose to bring the tails close to the bottom of the microscope dish. Once the low-melting agarose solidifies, the embryo medium (egg water) is added to the plate to keep the larvae alive during the imaging time. The zebrafish tail fin, at 3 dpf, is less than 50 μm thick [33]. In non-infected conditions, there are no macrophages in the area (Supplementary Figure S1). By introducing a localized infection, immune cell recruitment can be visualized.

Using the setup in Figure 1A, we performed local microinjection of fluorescently labeled *Mm* into the tail fin of 3 dpf transgenic zebrafish larvae expressing *mCherry-F* in the membrane of macrophages [29]. Microinjected larvae were then mounted for time-lapse imaging to study the early course of infection. At 30 min post-infection (mpi), fluorescent bacteria and macrophages were observed at the injection area, indicating that the injection triggered the host’s innate immune response (Figure 1B, arrow 1). Other macrophages were continuously recruited during the first 5 h of imaging the infection. Macrophages that performed phagocytosis of *Mm* displayed changes in morphology from a dendritic to a lobulated shape, as previously described to occur during macrophage polarization in zebrafish inflammation models [35] (Figure 1B, arrows 2 and 4, respectively). In addition, we observed cell–cell interactions between infected and non-infected macrophages. An example of such an event is shown at 93 mpi, where a non-infected cell approached an infected cell (Figure 1B, arrows 3 and 4, respectively). In less than 10 min, cytoplasmic membranes came in contact in a quick succession of events reminiscent of cell fusions previously observed during other infections [36], leading to the apparent fusion between these cells (Figure 1B, arrow 4′). During the time of imaging, some bacteria were not phagocytosed by macrophages. We hypothesize that they were captured by neutrophils or non-professional phagocytes, such as epithelial cells, as observed previously in the tail fin model [28,37]. In conclusion, the TFI and high-resolution live imaging approach allowed us to observe rapid phagocytosis of *Mm* by macrophages followed by dynamic interactions and apparent fusion with non-infected cells.

### 3.2. Mm Infection Rapidly Increases the LC3 Levels in Macrophages

**Figure 2:**
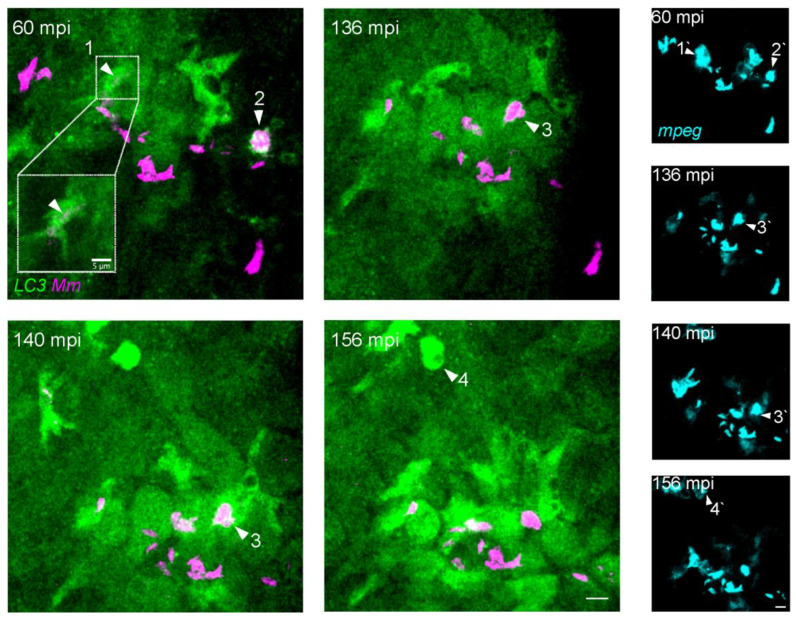
*Mm* infection induces an early LC3 increase in macrophages. Double transgenic (*mpeg1.1:mCherry-F/CMV:GFP-LC3*) zebrafish larvae, labeling macrophages (pseudo color cyan) and LC3 autophagy-related protein (green), were infected with 100 CFU of E2-crimson *Mm* (pseudo color magenta) following the TFI protocol. The figure shows the maximum projections of time-lapse crops of the injected area (Supplementary Video S3). Within the indicated time sequence from 60 and 156 mpi, macrophages are increasingly recruited to the site of infection. At 60 mpi, bacteria clusters are observed in association with the LC3 signal (arrowheads 1 and 2). The *mpeg1.1:mCherry-F* signal is shown separately to confirm the localization of the bacteria inside macrophages (arrowheads 1′ and 2′). The white square zooms in on arrowhead1, where a selection of the Z-stack is shown to unmasks the *Mm* cluster from the LC3 signal. Note that the high bacterial load of some macrophages at 60 mpi was comparable to the 93 mpi time point in Figure 1, suggesting slight differences in the injected dose. The level of LC3 signal in macrophages increases over time in infected (arrowheads 3/3′) and uninfected macrophages (arrowheads 4/4′). Scale bar: 10 μm.

To study autophagy pathway activation in response to *Mm* infection, we applied the TFI protocol in a double transgenic zebrafish line (*mpeg1.1:mCherry-F*/*CMV:GFP-LC3*), in which macrophages and the autophagy marker LC3 are fluorescently labeled [27,29]. In the time-lapse imaging, at 60 mpi, bacterial clusters were observed inside LC3-positive macrophages (Figure 2, arrowheads 1, 2, and 3). As macrophage numbers at the site of infection increased over time, so did their LC3-positive signal level (Figure 2, 156 mpi). We noted such augmented LC3 reporter gene activity in *Mm*-infected positive (Figure 2, arrowhead 3) as well as in uninfected macrophages (Figure 2, arrowhead 4), suggesting that the increase in LC3 reporter gene activity is a systemic effect of the response to infection. As a control, injection of PBS into the tail fin also elicited macrophage recruitment but did not lead to induction of LC3 reporter gene activation (Supplementary Figure S1, 60–180 mpi).

### 3.3. Intracellular Dynamics of LC3 Association with Mm

**Figure 3:**
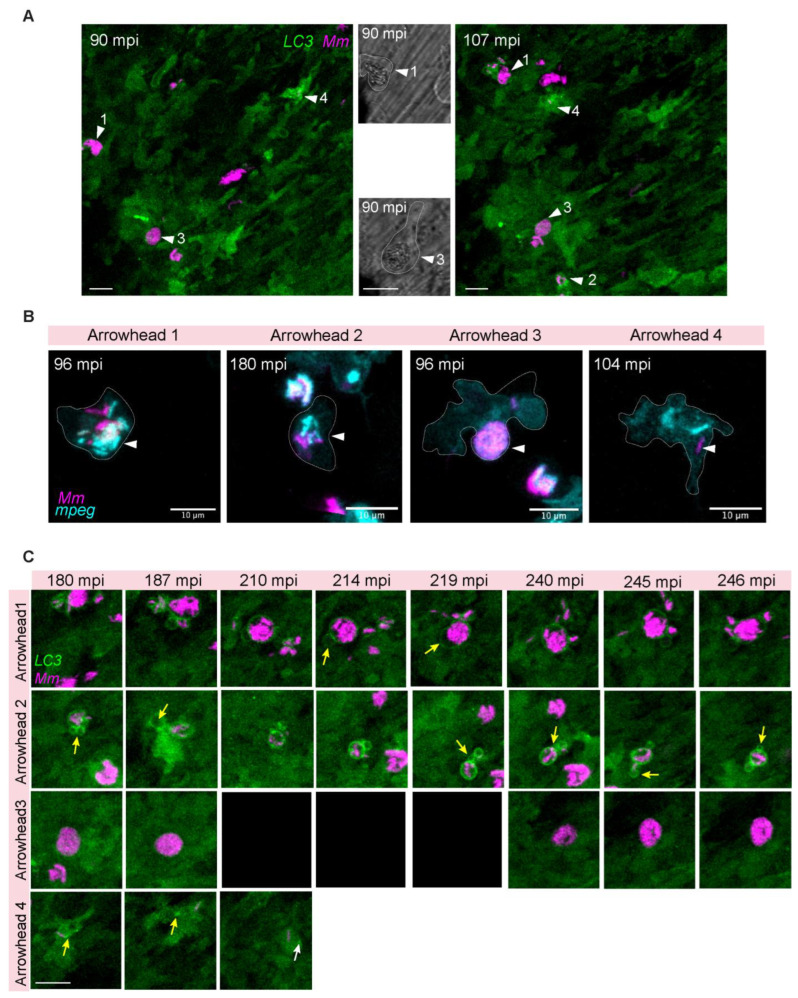
Intracellular dynamics of LC3 association with *Mm.* Double transgenic (*mpeg1.1:mCherry-F/CMV:GFP-LC3* zebrafish larvae, labeling macrophages (pseudo color cyan) and LC3 autophagy-related protein (green), were infected with 100 CFU of E2-crimson *Mm* (pseudo color magenta) following the TFI protocol. (**A**) Maximum projection of the entire time-lapse field of view of the injected area (Supplementary Video S4). At 90 mpi, LC3-positive, large (arrowhead 1), and small (arrowheads 2 and 4) *Mm* clusters are observed. At 107 mpi, an additional LC3-positive *Mm* cluster is within the field of view (arrowhead 2). The corresponding bright field (BF) images of 90 mpi show magnifications of the cells with intracellular clusters of bacteria, indicated by arrowheads 1 and 3. Cellular borders are outlined with dashed lines. (**B**) Zoomed-in areas corresponding to the arrowheads in A showing the *mpeg1.1:mCherry-F* signal (pseudo color cyan) to confirm the localization of *Mm* inside macrophages (**C**) Time series of zoomed-in areas corresponding to the arrowheads in A. Arrowhead 1 displays LC3-positive spacious containing a large *Mm* cluster, dynamically interacting with LC3-positive empty vesicles (yellow arrows). Arrowhead 2, LC3-positive compound vesicles associated with a small *Mm* cluster and undergoing vesicle fusion (yellow arrows 180–187 mpi). Arrowhead 3 points at an LC3-negative large *Mm* cluster that is observed over the entire time-lapse duration without any variation in size or distribution inside the cell compartments (the cell was out of focus due to adjustment of the tissue between 210–219 min)). Arrowhead 4, Compact *Mm*-containing vesicle inside a highly motile cell in close association with LC3 signal. LC3 bright puncta (white arrow) and tubular (yellow arrow) structures are adjacent to the bacteria. Scale bar: 10 μm.

The observed association of LC3 with *Mm* during early time points of infection (Figure 2) prompted us to further investigate the interaction between LC3 structures and bacterial clusters in the TFI setup. As before, we observed LC3 local induction at the site of infection within the first 2 h after infection (Figure 3A). We could distinguish at least four differentiated types of events starting from 90 mpi. These events describe *Mm* clusters inside macrophages (Figure 3B), where the observed LC3 signal levels were higher than the ubiquitous background of the transgenic zebrafish line. The first showed at 90 mpi around a large *Mm* cluster inside a macrophage with a low LC3 signal (Figure 3A,B, Arrowhead 1). At 107 mpi, the same cluster appeared fragmented into a big cluster inside an LC3-negative vacuole and small clusters in LC3-positive compact vesicles (Figure 3A, Arrowhead 1). At 187 mpi, we observed what we describe as LC3-positive compound vesicles (Figure 3C, Arrowhead 1). These compound vesicles were highly dynamic, sometimes in proximity to each other and sometimes making direct contact (Figure 3C, Arrowhead 1, 187–214 mpi). Some vesicles contained bacteria, and some were negative for bacterial signal. During 214–219 mpi (Figure 3C, Arrowhead 1, yellow arrows), we observed fusion between an empty and *Mm*-containing vesicle, increasing the size of the latter one. At 246 mpi, the LC3 signal dimmed, while the bacteria signal was still brightly fluorescent and more compact in structure than at earlier time points (Figure 3C, Arrowhead 1, e.g., compare 214 and 246 mpi).

The second type of event (Figure 3A,B, Arrowhead 2) was similar to the first in that LC3-positive compound vesicles were formed, but in this case, around a small group of bacteria. We observed fusion between the empty LC3-positive vesicles (Figure 3C, Arrowhead 2, 180–187 mpi, yellow arrows) and between empty and *Mm*-containing vesicles, increasing the size of the latter one (Figure 3C, Arrowhead 2, 219–240 mpi, yellow arrows). New vesicles were formed immediately after, in direct contact with *Mm*-containing vesicles (Figure 3C, Arrowhead 2, 245–246 mpi, yellow arrows). For this vesicle, no change in total bacterial load was observed.

The third type of event (Figure 3A,B, Arrowhead 3) is represented by a large phagocytosed LC3-negative *Mm* cluster. While LC3-negative, the *Mm* cluster was inside a vesicle (Figure 3A, Arrowhead 3, brightfield magnification). During the time the phagocytosed cluster was imaged, it never became LC3 positive, the bacterial load was continuously high, and the cell was barely motile (Figure 3C, Arrowhead 3, 180–246 mpi).

Finally, the fourth type of event apparent is represented by the formation of punctate LC3-signal around a small *Mm* cluster inside a highly LC3-positive macrophage (Figure 3A,B, Arrowhead 4). We followed this case while the infected macrophage moved inside the field of view (Figure 3C, Arrowhead 4). Puncta (210 mpi, white arrow) and tubular (180–187 mpi, yellow arrow) LC3 structures were located adjacent to a small bacterial cluster. The bacterial fluorescent intensity was stable over the time course of imaging.

In conclusion, while some *Mm* clusters are LC3-negative, dynamic LC3 vesicle interactions commonly occur around small and large *Mm* clusters, which included fusions between *Mm*-containing and empty vesicles. No evidence for bacterial killing in these vesicles was obtained by time-lapse imaging of the first 4 h after infection.

### 3.4. LC3-Positive Mm-Containing Vesicles Present Heterogeneous Dynamic Morphologies

**Figure 4:**
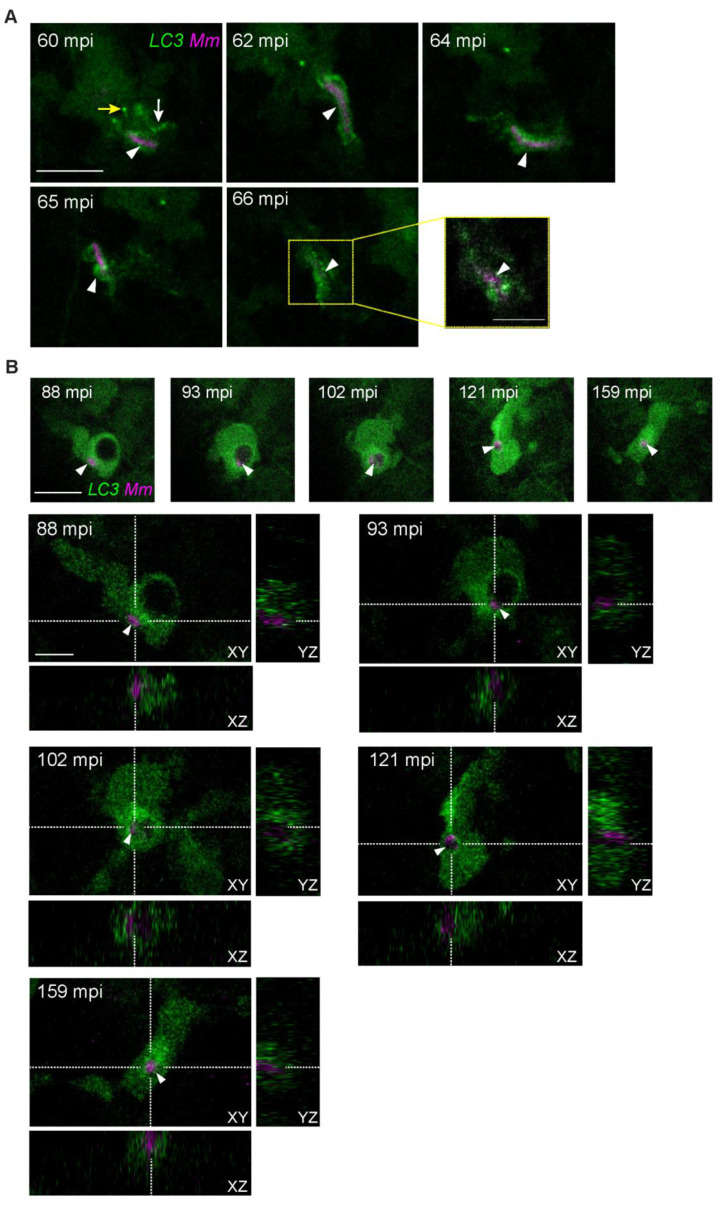
Heterogenous dynamic morphologies of LC3-positive *Mm*-containing vesicles. Transgenic (*CMV:GFP-LC3*) zebrafish larvae, labeling LC3 autophagy-related protein (green), were infected with 100 CFU of *Mm*-mCherry (pseudo color magenta) following the TFI protocol. (**A**) Maximum projection stills of an infected macrophage that reshapes an LC3-positive *Mm*-containing vesicle (arrowhead) during migration (60–65 mpi) (Supplementary Video S5). Note how the *Mm*-containing vesicle (arrowhead) is surrounded by puncta (yellow arrow) and tubular (white arrow) LC3-positive structures and subsequently changes into an elongated form (arrowhead, 62–64 mpi), then reshapes (65 mpi). A zoomed-in Z stack selection of the LC-positive structures at 66 min (outlined yellow) is shown to reveal the presence of *Mm*. (**B**) Orthogonal view of the complete Z-stacks, stills capturing the moment when an *Mm* cluster (white arrowhead) is incorporated into an LC3-positive spacious vesicle (88–93 mpi), after which the vesicle reshapes to a compact form (102–159 mpi) (Supplementary Video S6). Scale bars: 10 μm for all maximum projection stills in A and B, and 5 μm for the zoomed-in area in A and the orthogonal views in B.

The diversity of LC3 structures observed during time-lapse imaging of *Mm* infection made us look further into the stability of these structures over time. As an example, we tracked the sequence of events around a small bacterial cluster detected in an LC3-positive compact vesicle at 60 mpi (Figure 4A). We observed that this vesicle elongated along with the displacement of the macrophage through the tailfin tissue (Figure 4, 62–64 mpi). At 65 mpi, the vesicle reshaped into an LC3-positive compound vesicle type. As a result of the cell transversal displacement across the tail fin tissue, at 66 mpi, it seemed like the bacterial signal had disappeared. However, a close-up of a Z-stack selection uncovered that the *Mm* cluster was still present. As a second example, we detected over a sequence of 5 min the transition of a small *Mm* cluster into an LC3-positive spacious vesicle (Figure 4B, arrowhead 88–93 mpi, Z-stack projections, and orthogonal views). Subsequently, the spacious vesicle was reshaped into a compact form (Figure 4B, 102 mpi). An orthogonal view of the complete Z-stacks confirmed the *Mm*-containing vesicle constriction. The LC3-positive signal was gradually lost from the vesicle membrane (Figure 4B, 121–159 mpi). During this entire time of imaging, no change in the *Mm* fluorescent signal was detected. These results show that LC3-positive *Mm*-containing vesicles change their shape in a highly heterogeneous and rapid fashion, alternating between single versus compound states, rounded versus elongated morphologies, or spacious and compact morphologies. Furthermore, vesicles can also pass from an LC3-positive to an LC3-negative state without a detectable effect on the bacterial load.

### 3.5. LC3-Mm Dynamics Occur Independent of the Bacterial Dose and Is Detected in Phagocytes Disseminating Mm Infection

**Figure 5:**
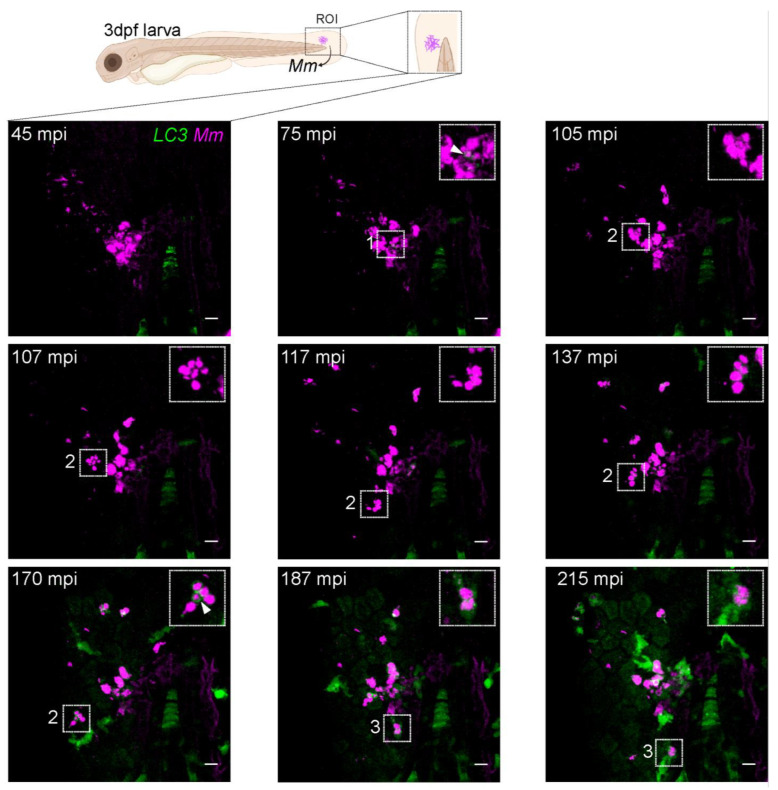
Early dissemination of *Mm* infection. Transgenic (*CMV:GFP-LC3*) zebrafish larvae, labeling LC3 autophagy-related protein (green), were infected with 300 CFU of *Mm*-mCherry (pseudo color magenta) following the TFI protocol. Maximum projection stills are shown of the entire time-lapse field of view of the injected area, with the ROI enlarged in the insets (Supplementary Video S7). At 75 mpi, the first LC3-positive *Mm*-containing vesicles are seen (inset, arrowhead). ROI 2 follows a big phagocytosed bacteria cluster displaced surrounding the core infection site while fragmented into smaller clusters. At 170 mpi, clear LC3-positive spacious vesicles engulf some previously fragmented clusters (inset, arrowhead). Area 2 follows a different LC3-positive *Mm* cluster. At 215 mpi, the phagocytic cell moves towards the proximity of the caudal vein area at the bottom of the field of view. Scale bar: 10 μm.

**Figure 6:**
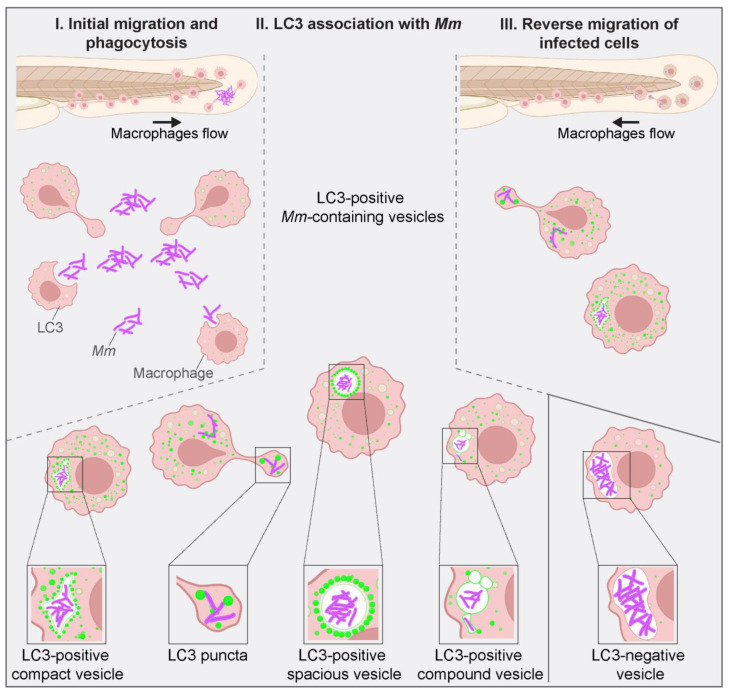
Schematic overview of LC3-associations with *Mm*-containing vesicles. Zebrafish in vivo imaging of *Mm* infection using the TFI protocol allowed us to distinguish between different LC3-vesicle membrane associations upon *Mm* infection. Macrophages are recruited to the site of infection at <30 mpi, and initial phagocytosis is observed at 30 mpi (Figure 1). At 60 mpi, LC3-vesicle membrane associations are visible in the proximity of bacterial clusters and colocalizing with them (Figure 4). The scheme illustrates the typically observed shapes: LC3-positive compact vesicle (Figure 2), LC3 puncta (Figure 4), LC3-positive spacious vesicle (Figure 5), and LC3-positive compound vesicles (Figure 3). Around 200 mpi, cells carrying bacteria inside LC3-positive vesicles were seen migrating away from the site of infection towards the caudal vein area, possibly disseminating the infection. Such motility was not observed for cells containing large LC3-negative bacterial clusters. Figure created with BioRender.

After we characterized diverse and fluctuating LC3-*Mm* associations (Figure 3 and Figure 4), we asked if a higher bacterial dose, 300 CFU, would present a different pattern or dynamics of LC3-associations. In a 5 h time-lapse sequence, we first observed small LC3-positive vesicles at the infection site by 75 mpi (Figure 5, 75 mpi, ROI 1 arrowhead). Compared with the previous data obtained with the lower infection dose, 100 CFU (Figure 2), there was no difference in the initial LC3 recruitment timing. Tracking of a large phagocytosed *Mm* cluster (Figure 5, ROI 2) showed that once phagocytosed, this cluster was fragmented into LC3-negative vesicles and displaced surrounding the primary infection site in ten minutes (Figure 5, 107–117 mpi, ROI 2). Subsequently, an LC3 signal appeared surrounding the fragmented clusters (Figure 5, 137 mpi, ROI 2) and developed over time into bright LC3-positive spacious vesicles enveloping three out of four fragmented *Mm* clusters (Figure 5, 170 mpi, ROI 2, arrowhead). This result confirms observations made with the lower dose of infection. As before, LC3-*Mm* associations were heterogeneous and dynamic. Notably, this diversity was more apparent due to a higher bacterial dose. All over the field of view, various LC3 structures transitioned from one pattern to another during the time-lapse. As an example, we traced a phagocyte carrying LC3-positive *Mm*-containing vesicles (Figure 5, ROI 3) while migrating through the tail fin tissue away from the site of infection (Figure 5, 187–300 mpi, ROI 3). This migration suggested early dissemination of infection into deeper tissues. In a schematic overview, we summarize the appearance of different types of LC3-*Mm* associations from the initial phagocytosis until infection dissemination (Figure 6).

## 4. Discussion and Conclusions

Autophagy is widely recognized as a crucial defense mechanism that represents a possible therapeutic target to enhance innate immunity against infectious diseases [38,39]. While many studies have provided evidence for a protective role of autophagy in infections with *Mtb* or other mycobacteria, it is still not well understood how these notorious pathogens interact with the autophagy machinery [7,21,40,41,42,43,44]. First, the differential roles of autophagy versus autophagy-related pathways, such as LAP, in mycobacterial host defense remain to be dissected. Second, more research is needed into the virulence mechanisms that mycobacteria have evolved to counterattack the host autophagic machinery or use it for their own benefit [39,45,46]. Third, most observations of the autophagic targeting of pathogens rely on cell-based experimental systems that do not fully reproduce the in vivo infection context. To address this limitation in mycobacterial autophagy research, here we exploited a zebrafish infection model that enables live imaging of the interaction between mycobacteria and the autophagy machinery in infected macrophages, from the onset of phagocytosis up to the dissemination of mycobacteria away from the initial infection site.

The TFI technique, wherein *Mm* is injected into the thin tissue of the zebrafish tail fin, permitted visualization of macrophage–*Mm* interactions minutes after infecting the samples. The host immune response was denoted by macrophage recruitment to the site of infection, one of the earliest infected cell types [47,48]. Initial phagocytosis was spotted at 30 mpi [28] and in agreement with previous work, we observed the recruitment of several macrophages during the first hours of infection [37]. While neutrophils also contribute to *Mm* phagocytosis [37], these cells were not labeled in our present study. We focused on macrophages as these are the main drivers of mycobacterial pathogenesis [49]. Adding to the observation that macrophages are responsible for efferocytosis of dead infected cells, we spotted cell–cell interactions between infected and non-infected macrophages, reminiscent of cell fusion. This suggests that bacteria-induced cell fusion may contribute to mycobacterial pathogenesis, similar to what has been described for *Burkholderia pseudomallei*, which induces cell fusion to spread intracellularly, escaping extracellular host defenses [36].

We determined the activation of the autophagy machinery, reported by the GFP-LC3 signal. Within the first two hours of infection, we noted increased GFP-LC3 signal intensity in the macrophages recruited to the site above the basal background signal of the transgenic zebrafish line. This response appeared to be systemic as it occurred in infected and non-infected bystander macrophages, and rather than reflecting endogenous LC3 levels, it might reflect infection-induced transcriptional activation of the GFP-LC3 reporter or increased GFP-LC3 protein stability. At the subcellular level, infected cells between 1–4 hpi showed different types of higher intensity GFP-LC3 signal around *Mm*, sometimes appearing as puncta in association with *Mm* but also as ring-like signals on vesicles containing *Mm*, all detected between one and several hours. The punctate GFP-LC3 vesicles, not containing bacteria, had previously been considered non-specific, and bacteria-containing GFP-LC3-positive *Mm*-containing vesicles had only observed after one day [50]. Our present work shows that the autophagy machinery captures *Mm* already in the first hours after infection in vivo, underscoring its relevance for early mycobacterial pathogenesis [28].

By examining the in vivo dynamics of LC3-*Mm* associations, we could distinguish four phenotypes: LC3-positive puncta, spacious, compact, and compound vesicles. These vesicles were not only remarkably heterogeneous but could dynamically change their morphology. Such dynamics were observed when phagocytes were migrating around the site of infection as well as during reverse migration. Compound vesicles emerged by fusions between *Mm*-containing and empty vesicle fusions, and subsequentially broke up and regrouped. In addition, rounded vesicles elongated during macrophage cell migration, spacious vesicles condensed to enclose *Mm* tightly, and LC3 signal could be transiently gained and lost on these vesicles. Together, these observations indicate that different vesicle phenotypes can appear in the same cell type simultaneously or sequentially, and that there is high plasticity with different vesicle types transitioning from one phenotype into another. The vesicle heterogeneity in zebrafish matches in vitro results obtained using *Mtb* and human iPSC-derived macrophages [50]. This study describes the formation of LC3 tubulovesicular structures (LC3-TVS) at 2 hpi [50]. LC3-TVS showed similar morphology and dynamic behavior to our four types of phenotypes. Therefore, these results provide essential cross-validation to each other, showing that LC3 response to mycobacteria is conserved between *Mtb* and *Mm* infections, between human and zebrafish macrophages, and between in vitro and in vivo models.

The LC3-TVS observed in *Mtb*-infected iPSC-derived macrophages were identified as autophagosomes, supported by transmission electron microscopy data [50]. In the zebrafish TFI model, the presence of *Mm* in autophagosomes has also been detected, albeit at later stages of infection than the one studied here [28]. In our current study of the early infection stages, we cannot discard the possibility that LC3-positive structures resulted from the occurrence of LAP activation. We observed *Mm* clusters positive for LC3 at <90 mpi, faster than the mentioned in vitro data. In LAP, phagocytosis induces LC3 conjugation to phagosomes, which can occur more rapidly than the autophagy response that predominantly targets mycobacteria after they have translocated from phagosomes into the cytosol [6,51]. In our time-lapse imaging, we have not been able to ascertain if cytosolic escape preceded LC3 recruitment and thus cannot exclude LAP activation induced by *Mm* infection. The possible contribution of LAP is consistent with results showing that LAP is an important defense mechanism in *Mtb* infection that has to be counteracted by *Mtb* virulence [19].

Finally, an open question remains: What is the fate of *Mm* captured by LC3-positive vesicles? In our time-lapse series, we have not observed evidence of bacterial killing, neither in LC3-negative nor in LC3-positive vesicles. Our time lapse data indicate that *Mm* can withstand the environment of LC3-positive vesicles for at least several hours. In addition, we observed how macrophages carrying LC3-positive *Mm*-containing vesicles migrated away from the infection site, suggesting early infection dissemination. These results support the hypothesis of autophagic compartments as a niche for bacteria replication and the success of infection [46]. However, evidence also supports the host-protective role of the autophagic mechanism in the zebrafish model. Specifically, we have shown that the selective autophagy receptors, p62, and Optineurin, as well as the autophagy modulator Dram1, are required for effective host defense against *Mm* [21,24,25]. Together, the zebrafish studies suggest that *Mm* can evade autophagic killing to a certain extent yet is ultimately vulnerable to the autophagic host defenses.

In conclusion, due to the zebrafish transparency and application of the TFI technique, we could observe intracellular processes during the early progression of infection with comparable clarity to what has been achieved with in vitro models [50]. This in vivo study revealed heterogeneous and dynamic interactions between mycobacteria and the autophagy machinery, providing a framework for further research into this process’s underlying mechanisms and potential drug targeting.

## Data Availability

All data are based on microscopic imaging videos, which are included as supplementary data files.

## Supplementary Materials

The following supporting information can be downloaded at: https://www.mdpi.com/article/10.3390/biology12060817/s1, Video S1: Figure 1a, Video S2: Figure 1b, Video S3: Figure 2, Video S4: Figure 3, Video S5: Figure 4a, Video S6: Figure 4b, Video S7: Figure 5.
