# Supplementary material for: Using Zebrafish to Dissect the Interaction of Mycobacteria with the Autophagic Machinery in Macrophages
Source: Biology (Basel). 2023 Jun 4;12(6):817. doi: 10.3390/biology12060817 (PMC10295493; doi:10.3390/biology12060817)
Supplement: Supplementary file 1 [file biology-12-00817-s001.zip › Supplementary Figure 1.pdf]

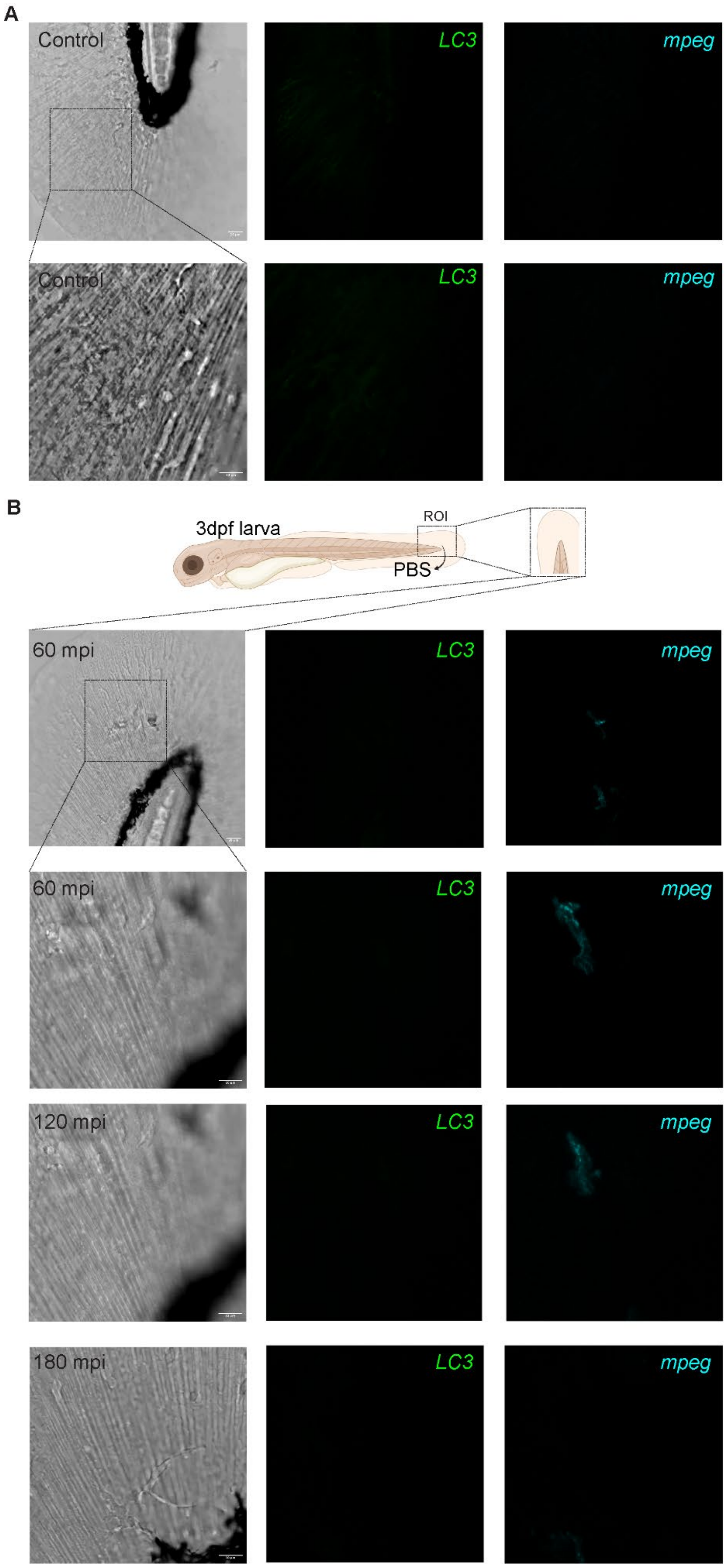

Supplementary figure 1: Confocal laser scanning microscopy (CLSM) live imaging of non-injected and PBS- injected tail fins. Double transgenic (CMV:GFP-LC3/ mpeg1.1:mCherry-F) 3dpf zebrafish larvae were used, labeling LC3 autophagy-related protein (green) and macrophages (pseudo color cyan). (A) Non-injected controls tail fin live imaging. Tail fin does not contain macrophages or GFP-LC3 signal activation. (B) Schematic of the PBS-injected controls depicting the region of interest (ROI) and live imaging from 60 to 180 mpi. The time sequence shows migration of macrophages to the site of injection but without GFP-LC3 activation.
